# Relapse-free survival with adjuvant dabrafenib/trametinib therapy after relapse on a prior adjuvant CPI in BRAF V600-mutated stage III/IV melanoma

**DOI:** 10.1093/oncolo/oyae289

**Published:** 2024-11-19

**Authors:** Jeffrey Weber, Waqas Haque, Svetomir N Markovic, April K S Salama, Inderjit Mehmi, Ryan J Sullivan, Yana G Najjar, Alexander C J van Akkooi, Alexander M Menzies, Georgina V Long, Amelia M Taylor, John Haanen, Lisanne P Zijlker, Keith L Davis, Siddharth Karanth, Deborah Norton, Lucy Connolly

**Affiliations:** Laura and Isaac Perlmutter Comprehensive Cancer Center, NYU Grossman School of Medicine, NYU Langone, New York, NY 10016, United States; Department of Medicine, NYU Grossman School of Medicine, NYU Langone, New York, NY 10016, United States; Division of Medical Oncology, Department of Oncology, Mayo Clinic Comprehensive Cancer Center, Rochester, MN 55905, United States; Division of Medical Oncology, Department of Medicine, Duke University School of Medicine, Durham, NC 27710, United States; The Angeles Clinic and Research Institute, Cedars Sinai, Los Angeles, CA 90025, United States; Center for Melanoma, Massachusetts General Hospital, Boston, MA 02114, United States; Clinical and Translational Research Center, UPMC Hillman Cancer Center, Pittsburgh, PA 15232, United States; Melanoma Surgical Oncology, Melanoma Institute Australia, Wollstonecraft, Sydney, NSW 2065, Australia; Faculty of Medicine and Health, University of Sydney, Camperdown, Sydney, NSW 2050, Australia; Department of Melanoma and Soft Tissue Sarcoma, Division of Surgical Oncology, Netherlands Cancer Institute – Antoni van Leeuwenhoek, 1066 CX Amsterdam, The Netherlands; Faculty of Medicine and Health, University of Sydney, Camperdown, Sydney, NSW 2050, Australia; Medical Oncology, Melanoma Institute Australia, Wollstonecraft, Sydney, NSW 2065, Australia; Medical Oncology, Royal North Shore Hospital, Northern Sydney Cancer Centre, St Leonards, NSW 2065, Australia; Department of Cancer Medicine, Mater Hospital, North Sydney, NSW 2060, Australia; Faculty of Medicine and Health, University of Sydney, Camperdown, Sydney, NSW 2050, Australia; Medical Oncology, Melanoma Institute Australia, Wollstonecraft, Sydney, NSW 2065, Australia; Medical Oncology, Royal North Shore Hospital, Northern Sydney Cancer Centre, St Leonards, NSW 2065, Australia; Medical Oncology, Mater Hospital, North Sydney, NSW 2060, Australia; Medical Oncology, Melanoma Institute Australia, Wollstonecraft, Sydney, NSW 2065, Australia; Division of Medical Oncology, Netherlands Cancer Institute – Antoni van Leeuwenhoek, 1066 CX Amsterdam, The Netherlands; Medical Oncology (Service d’oncologie médicale), Centre Hospitalier Universitaire Vaudois (CHUV), 1005 Lausanne, Switzerland; Division of Surgical Oncology, Netherlands Cancer Institute – Antoni van Leeuwenhoek, 1066 CX Amsterdam, The Netherlands; Medical Oncology, Royal North Shore Hospital, Northern Sydney Cancer Centre, St Leonards, NSW 2065, Australia; Real-World Data and Analytics, RTI Health Solutions, Research Triangle Park, NC 27709, United States; Melanoma US Medical Team, Novartis Pharmaceuticals Corporation, East Hanover, NJ 07936, United States; Real World Evidence Solutions, CONEXTS, Novartis Ireland Ltd., Dublin 4, D04 NN12, Ireland

**Keywords:** BRAF mutation, melanoma, dabrafenib, trametinib, relapse-free survival

## Abstract

**Background:**

In BRAF-mutated high-risk melanoma, targeted therapy (BRAF/MEK inhibitors) and checkpoint inhibitor (CPI) immunotherapy have durable benefits as first-line (1L) adjuvant therapy. Based on differing action mechanisms of BRAF/MEK inhibitors and CPI immunotherapies, there is interest in evaluating the activity of 2L adjuvant targeted therapy in decreasing the risk of subsequent recurrence after repeat resection following relapse on/after 1L adjuvant CPI.

**Patients and methods:**

This was a retrospective review of BRAF V600-mutated resected stage III/IV melanoma patients in the United States, Australia, and The Netherlands who received 1L adjuvant CPI immunotherapy, relapsed locoregionally/distantly, were again resected to no evidence of disease, and received dabrafenib/trametinib (dab/tram) as 2L adjuvant therapy. The primary endpoint was relapse-free survival (RFS) from initiation of 2L adjuvant dab/tram (RFS-2), analyzed via Kaplan-Meier methods.

**Results:**

Thirty-eight patients were included (median age 50 years, 63% male, 87% stage III, median follow-up 19 months from 2L dab/tram initiation). Median dab/tram duration was 10.1 months (range: 1 day–22.7 months), with half discontinuing due to progression or adverse events. Median (95% CI) RFS-2 was 18.9 (14.9–28.1) months, with 91%, 81%, and 58% remaining relapse-free at 6, 12, and 18 months, respectively. Most patients remained distant metastasis-free at 6, 12, and 18 months (97%, 85%, and 71%, respectively). Two patients were deceased at the last follow-up, with 97% alive at 18 months.

**Conclusions:**

Over 80% of patients remained relapse- and metastasis-free at 12 months after 2L dab/tram initiation, with only 2 deaths observed. Dab/tram appears to have activity in the 2L adjuvant setting, although more follow-up is required.

**Implications for practice**

Targeted therapy (BRAF/MEK inhibitors dabrafenib/trametinib [dab/tram]) and CPI immunotherapies have shown durable clinical benefit as 1L adjuvant therapy in patients with BRAF-mutant resected stage III melanoma; however, a substantial proportion of these patients will eventually relapse. In this study dab/tram appears to have activity in the 2L adjuvant setting, although more follow up is required. Over 80% of patients remained relapse- and metastasis-free at 12 months after 2L dab/tram initiation, with only 2 deaths observed, and 97% of patients alive at 18 months. These findings may help inform clinicians currently managing high-risk melanoma patients who relapse after adjuvant CPI therapy.

## Background

Both targeted therapy (the BRAF- plus MEK-inhibitor combination of dabrafenib plus trametinib [dab/tram]) and checkpoint inhibitor (CPI) immunotherapies (ipilimumab, nivolumab, or pembrolizumab) have shown durable clinical benefit as first-line (1L) adjuvant therapy in BRAF-mutant resected stage III melanoma.^1-4^ These therapies are now considered the standard of care for adequately resected high-risk melanoma and represent a significant advancement in the treatment of this disease. Despite a proven relapse-free survival (RFS) benefit with these therapies, a substantial proportion of patients with resected advanced melanoma receiving 1L adjuvant dab/tram or CPI immunotherapy will relapse; optimal clinical management of these patients following relapse remains undefined.^5^

Two retrospective studies have recently assessed patterns of recurrence and subsequent treatment of patients with resected advanced melanoma who underwent 1L adjuvant targeted therapy or CPI immunotherapy.^6,7^ Findings from these studies, as well as several prospective trials,^3,4,8^ suggest that a majority of relapses on adjuvant immunotherapy occur within the first 12 months, whereas relapse is rare during the first 12 months on adjuvant BRAF-targeted therapy. Another observation from these studies is that approximately half of the recurrences are in patients who loco-regionally recur and can often be resected to no evidence of disease (NED) again. Given these findings and based on an understanding of the differing mechanisms of action of BRAF/MEK inhibitors and CPI immunotherapies, there is an interest in evaluating the activity of second-line (2L) adjuvant targeted therapy with dab/tram to decrease the risk of subsequent recurrence in patients with repeat surgical resection following locoregional or distant relapse on or after 1L adjuvant CPI immunotherapy. The objective of this study was to therefore examine and report relevant clinical outcomes of these patients in the 2L setting. Such data may help inform the direction of future clinical trials and provide additional insights for healthcare providers currently managing patients with double-relapsed BRAF-mutant melanoma.

## Methods

**Figure 1.**
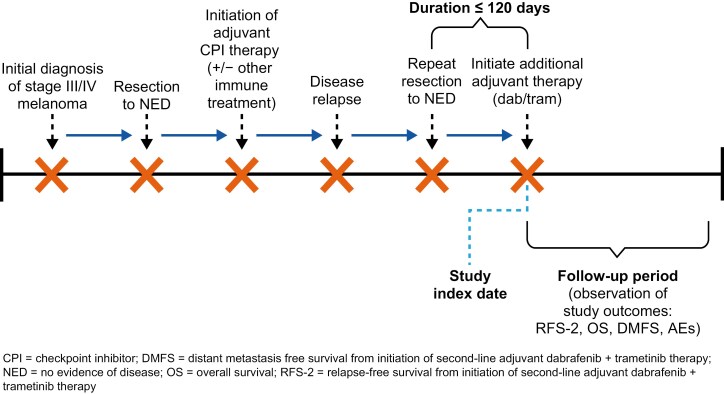
Study design schema. Abbreviations: CPI, checkpoint inhibitor; DMFS, distant metastasis-free survival from initiation of second-line adjuvant dabrafenib + trametinib therapy; NED, no evidence of disease; OS, overall survival; RFS-2 = relapse-free survival from initiation of second-line adjuvant dabrafenib + trametinib therapy

This was a retrospective, multicenter chart review study of BRAF-mutated stage III/IV melanoma patients in the United States, Australia, and The Netherlands. All patients from the study sites’ caseloads meeting the following criteria were included in the study: (1) at least 18 years of age at diagnosis of stage III/IV melanoma; surgically resected to NED; (2) local or central assay-positive for tumor BRAF V600E/K mutation; (3) received 1L adjuvant CPI immunotherapy alone or in combination with any other immune treatment; (4) relapsed on or off 1L adjuvant treatment and again rendered disease-free after repeat surgical resection; (5) received additional (2L) adjuvant dab/tram combination therapy after repeat resection to NED. The date of initiating 2L adjuvant dab/tram defined the study index date. Patients previously administered any BRAF- or MEK-inhibiting therapy, who had more than 120 days duration between repeat resection and initiation of 2L adjuvant dab/tram therapy, or who received any intervening antitumor therapy between relapse to 1L adjuvant CPI immunotherapy and initiation of 2L adjuvant dab/tram therapy were excluded. Figure 1 presents a graphical depiction of the study design and schema. Chart reviews and data entry were conducted by site investigators or delegated clinical staff, who had direct authorized access to their patients’ medical records, from June 2022 through November 2023. All data were captured in a secure, online case report form that included numerous edit and logic checks to facilitate real-time data cleaning.

In the population described above, the primary endpoint assessed was RFS-2, defined as relapse-free survival after receipt of 2L dab/tram combination therapy following relapse on or after 1L adjuvant CPI immunotherapy and repeat resection to NED as described in Figure 1. Duration of RFS-2 was calculated as the time (in months) from dab/tram initiation until the earliest clinician-documented disease progression, death, or the start of a new line of anticancer treatment due to disease progression. Secondary endpoints included overall survival (OS) and distant metastasis-free survival (DMFS). OS was measured as time (in months) from initiation of 2L dab/tram adjuvant therapy until death due to any cause. DMFS was measured as time (in months) from initiation of 2L dab/tram adjuvant therapy until first observation of disease progression with distant metastasis among patients who were initially at stage III disease and recurred locoregionally or distantly with repeat resection to NED. Finally, selected adverse events (AEs) of interest were examined as the overall proportion of patients with an AE during exposure to 2L adjuvant dab/tram therapy. All analyses were descriptive in nature and conducted using SAS Studio statistical software. Event time endpoints (RFS-2, OS, and DMFS) were analyzed using Kaplan-Meier methods in which patients without an event were censored at the last available follow-up, defined as the last known clinic visit or last known contact with the patient (if occurred outside the clinic setting). Institutional review board (IRB) approvals were obtained at each center prior to data collection.

## Results

A total of 38 patients were included in the study (Table 1). The median age was 50 years, and 63.2% (24 patients) were male, with a median follow-up duration of 38 months from initial stage III/IV melanoma diagnosis and 19 months from initiation of 2L adjuvant dab/tram therapy. The median time from initial stage III/IV melanoma diagnosis until the index date was 16.4 months, and 86.8% (33 patients) had stage III disease at the index date. Among the 5 patients with stage IV disease at the index date, 3 patients had M1a metastasis (lymph nodes, skin/subcutaneous tissue, soft tissue), 1 patient had M1b metastasis (lung), and 1 patient had M1c metastasis (gall bladder).

**Table 1.** Patient demographics and clinical characteristics.

Total patients, *n* (%)	38	100%
Age at stage III/IV melanoma diagnosis, years		
Median	50	
Min, max	20	75
Age at additional adjuvant therapy with dabrafenib + trametinib (index date), years		
Median	52	
Min, max	21	76
Sex, *n* (%)		
Female	13	34.2%
Male	24	63.2%
Non-binary	1	2.6%
Predominant race, *n* (%)		
White	27	71.0%
Don’t know/not provided	11	30.0%
Ethnicity, *n* (%)		
Not Hispanic or Latina/Latino	27	71.0%
Don’t know	11	30.0%
Country, *n* (%)		
United States	15	39.47%
Australia	11	28.95%
Netherlands	12	31.58%
Duration (months) of follow-up		
From stage III/IV melanoma diagnosis		
Median	38	
Min, max	10	106
From index date		
Median	19	
Min, max	0	63
Disease stage at index date, *n* (%)		
Stage III	33	86.8%
Stage IV	5	13.2%
Don’t know	0	0.0%
Time from stage III/IV melanoma diagnosis to index date, in months		
Mean (SD)	22.9	20.2
Median	16.4	
Min, max	3.2	86.4
Metastatic site(s) involved at index date for patients with stage IV disease at diagnosis, *n* (%)	*n* = 5 (100%)	
M1a (lymph nodes, skin/subcutaneous tissue, soft tissue)	3	60.0%
M1b (lung)	1	20.0%
M1c (gallbladder)	1	20.0%
ECOG performance status at index date, *n* (%)		
0 (Asymptomatic)	32	84.2%
1 (Symptomatic, completely ambulatory)	5	13.2%
Not recorded at the time of index date	1	2.6%
Vital status at the end of follow-up, *n* (%)		
Alive	36	94.7%
Deceased	2	5.3%

Abbreviations: ECOG, Eastern Cooperative Oncology Group; SD, standard deviation.

Nivolumab alone was the most common CPI immunotherapy used in 1L adjuvant treatment (73.7% of patients); the median time to relapse after initiating 1L adjuvant CPI immunotherapy was 8.0 months, with 23 patients (60.5%) having relapsed within 12 months (Table 2). Median time to dab/tram initiation after repeat resection to NED was 3.9 weeks (range: 0.7–12.3 weeks), with half of patients (50%) having discontinued dab/tram prematurely due to progression (5 patients) or AEs/toxicity (14 patients) by last follow-up. Among the 14 patients who had discontinued due to AEs or toxicity, the most common event was fever/chills (8 patients); no patient discontinued dab/tram due to death. The median (95% confidence interval [CI]) dab/tram therapy duration, estimated via the Kaplan-Meier method, was 11.8 (8.7–12.3) months.

**Table 2.** Treatment characteristics.

Total patients, *n* (%)	38	100%
First adjuvant CPI therapy following initial surgical resection		
Nivolumab	28	73.7%
Pembrolizumab	6	15.8%
Nivolumab + ipilimumab	3	7.9%
Ipilimumab	1	2.6%
Additional chemo/immune therapies received in combination with adjuvant CPI therapy	0	0.0%
Time (months) to initiation of first adjuvant CPI therapy following initial surgical resection		
Mean (SD)	8.5	18.6
Median	1.9	
Min, max	0.5 *(14 days)*	72.5
**Time (months) to relapse after starting initial adjuvant CPI therapy**		
Mean (SD)	11.3	10.4
Median	8.0	
Min, max	1.4	49.8
Relapsed within ≤12 months, *n* (%)	23	60.5%
Relapsed after >12 months, *n* (%)	15	39.5%
Time (weeks) to additional surgical resection after relapse to initial adjuvant CPI therapy		
Mean (SD)	5.4	4.8
Median	4	
Min, max	0 *(0 days)*	21.9
Time (weeks) to dabrafenib + trametinib adjuvant therapy after additional surgical resection w/no evidence of disease		
Mean (SD)	5	3
Median	4	
Min, max	0.7 *(5 days)*	12.3
Dabrafenib + trametinib status at end of follow-up, *n* (%)		
Completed planned course of treatment	8	21.1%
Discontinued prematurely^a^	19	50.0%
Ongoing (as of last medical record)	11	28.9%
Don’t know	0	0.0%
Kaplan-Meier estimate of time (months) on dabrafenib + trametinib adjuvant therapy		
Mean (SE)	10.6	0.9
Median (95% CI)	11.8	(8.7-12.3)

^a^Fourteen patients discontinued prematurely due to adverse events, toxicity, or tolerability issues, and 5 patients discontinued prematurely due to disease progression.

Abbreviations: CPI, checkpoint inhibitor; SD, standard deviation.

**Figure 2.**
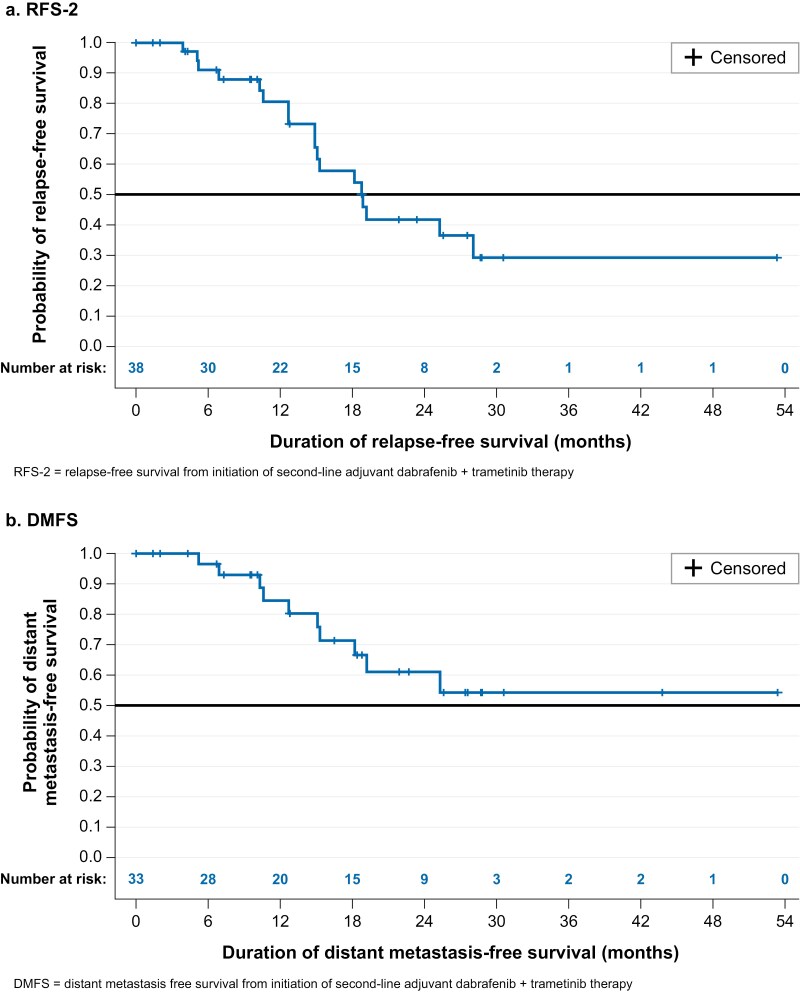
Kaplan-Meier survival curves. (A) RFS-2. (B) DMFS. Abbreviations DMFS, distant metastasis-free survival from initiation of second-line adjuvant dabrafenib + trametinib therapy; RFS-2, relapse-free survival from initiation of second-line adjuvant dabrafenib + trametinib therapy.

Median (95% confidence interval [CI]) RFS-2 was 18.9 (14.9–28.1) months, with 91.0% and 80.6% of patients remaining relapse-free at 6 and 12 months, respectively (Table 3, Figure 2a); at 18 months, RFS-2 (95% CI) dropped to 57.9%. Most of the 33 patients with stage III disease at the index date remained distant metastasis-free at 6 months (97%) and 12 months (85%) (Table 3, Figure 2b). Two patients were deceased at the last follow- up (both deaths were melanoma-related), with nearly all patients (97%) still alive at 18 months; median OS was not reached. Pyrexia (57.9%), chills (39.5%), fatigue or asthenia (28.9%), arthralgia (21.1%), headache (21.1%), and nausea (21.1%) were the most commonly observed AEs during dab/tram treatment (Table S1, Supplemental Material).

**Table 3.** RFS-2 and DMFS from the study index date, Kaplan-Meier estimates

	RFS-2		DMFS	
Total patients, *n* (%)	38	100.0%	33	100%
*N* (%) with event	18	47.4%	10	30.3%
*N* (%) censored	20	52.6%	23	69.7%
Median time to event (95% CI)	18.9	(14.9-28.1)	NR	(15.3—NR)
RFS-2/DMFS rate, *n* (%)				
At 6 months	91.0%	(74.8%-97.0%)	96.6%	(77.9%-98.5%)
At 12 months	80.6%	(61.6%-90.9%)	84.5%	(63.6%-93.9%)
At 18 months	57.9%	(37.5%-73.7%)	71.4%	(48.9%-85.3%)

Abbreviations: CI, confidence interval; DMFS, distant metastasis-free survival from initiation of second-line adjuvant dabrafenib + trametinib therapy; RFS-2, relapse-free survival from initiation of second-line adjuvant dabrafenib + trametinib therapy.

## Discussion/conclusion

The landscape for 1L adjuvant therapy in patients with resected high-risk, advanced melanoma has improved significantly over the past decade with the advent of BRAF/MEK targeted therapy such as dab/tram or CPI immunotherapies as the new standards of care with demonstrated durable clinical benefit as measured by RFS and DMFS. Based on results from the COMBI-AD trial observing that a majority of relapses to 1L adjuvant CPI immunotherapy occur within 12 months of initiation, with only 7% of 1L adjuvant dab/tram recipients relapsing within 12 months,^9^ there has been increased interest in examining clinical outcomes of patients relapsed to 1L adjuvant CPI immunotherapy who then received additional (2L) dab/tram adjuvant therapy following repeat resection to NED. A very recent observational study of patients with BRAF mutated melanoma that recurred after adjuvant PD-1-based immunotherapy reported significantly improved RFS-2 with dab/tram as a second adjuvant treatment vs RFS-2 with no second adjuvant treatment, but at the cost of toxicity.^10^

In the small cohort of such patients examined here, we show that clinical outcomes were favorable in the short-term while patients were on dab/tram treatment in the 2L adjuvant setting, with more than 80% of patients remaining relapse- and metastasis-free for at least 12 months after treatment initiation. In the longer term, effectiveness appeared to decrease with 58% remaining relapse-free at 18 months. However, only 2 deaths were observed and 97% of patients remained alive at 18 months. Finally, we observed a somewhat higher rate of premature discontinuation of dab/tram due to AEs or toxicity (37%) as compared with the COMBI-AD trial (26%),^9^ but there were no new or unexpected AEs observed for dab/tram therapy in this cohort of patients.

This study has several limitations inherent in retrospective chart reviews. First, the study captured a convenience sample of patients with stage III/IV resected BRAF-mutated melanoma from high-volume academic institutions that may not be generalizable to the broader population of these patients. Second, in retrospective studies, imaging assessments are not necessarily completed on a standardized schedule (for purposes of identifying further disease recurrence), as commonly used in clinical trials. For this reason, findings regarding the endpoints of RFS-2 and DMFS may not be directly comparable with those observed in clinical trials. Finally, findings presented here represent the clinical observations of patients treated at the institutions participating in this study; results may therefore not be generalizable to the broader population of patients with BRAF-mutant high-risk melanoma who have received 2L adjuvant dab/tram therapy.

In conclusion, targeted therapy with dab/tram combination treatment appears to have activity in the 2L adjuvant setting, although more follow up is required to confirm. In this study, more than 80% of patients remained relapse- and metastasis-free at 12 months after initiation of 2L adjuvant dab/tram, which is lower than that observed in the COMBI-AD trial for 1L adjuvant dab/tram.^9^ Two deaths were observed, and 97% of patients were alive at 18 months. There appears to be no new or unexpected AE signals observed for dab/tram therapy in the 2L adjuvant setting. Future studies should include follow-up to subsequent relapse or sustained disease response, with data capturing timing and sites of subsequent relapse, as well as additional treatments (eg, additional surgical resection, additional adjuvant or salvage therapy) applied in the 3L setting and beyond. Such data might help inform clinicians currently managing relapsed high-risk melanoma patients who may have failed one or even two lines of prior adjuvant therapy and may also help inform the direction of future clinical trials.

## Supplementary material

Supplementary material is available at *The Oncologist* online.

oyae289_suppl_Supplementary_Material

## Data Availability

Patient-level data for this study were collected via chart review at the study sites involved; due to the data privacy requirements described in the site-specific IRBs, the data collected are not publicly available.

## Statement of patient and public involvement

It was not appropriate or possible to involve patients or the public in the design, or conduct, or reporting, or dissemination plans of our research.
