# Supplementary material for: Relapse-free survival with adjuvant dabrafenib/trametinib therapy after relapse on a prior adjuvant CPI in BRAF V600-mutated stage III/IV melanoma
Source: Oncologist. 2024 Nov 19;30(3):oyae289. doi: 10.1093/oncolo/oyae289 (PMC11954497; doi:10.1093/oncolo/oyae289)
Supplement: oyae289_suppl_Supplementary_Material [file oyae289_suppl_supplementary_material.docx]

# SUPPLEMENTAL (ONLINE-ONLY) TABLES

#

| **Table S-1. Adverse Events During 2L Adjuvant Dabrafenib + Trametinib** | | | | | | | |
| --- | --- | --- | --- | --- | --- | --- | --- |
| **Adverse Event** | **Incident AE** | | **Number of Times AE Experienced** | | | | |
|  | N | % | Mean | SD | Median | Min | Max |
| Pyrexia | 22 | 57.9% | 4.7 | 13.1 | 1 | 1 | 63 |
| Fatigue or asthenia | 11 | 28.9% | 9.8 | 18.8 | 1 | 1 | 63 |
| Chills | 15 | 39.5% | 7.4 | 16.5 | 1 | 1 | 63 |
| Influenza-like illness | 4 | 10.5% | 2.5 | 3.0 | 1 | 1 | 7 |
| Headache | 8 | 21.1% | 8.8 | 16.8 | 3 | 1 | 50 |
| Rash | 7 | 18.4% | 3.7 | 7.2 | 1 | 1 | 20 |
| Nausea | 8 | 21.1% | 9.5 | 16.5 | 4 | 1 | 50 |
| Diarrhea | 4 | 10.5% | 3.3 | 2.2 | 3 | 1 | 6 |
| Vomiting | 5 | 13.2% | 4.2 | 2.9 | 4 | 1 | 8 |
| Constipation | 2 | 5.3% | 1.0 | 0.0 | 1 | 1 | 1 |
| Increase in ALT | 2 | 5.3% | 1.0 | 0.0 | 1 | 1 | 1 |
| Increase in AST | 2 | 5.3% | 1.0 | 0.0 | 1 | 1 | 1 |
| Cough | 0 | 0.0% | --- | --- | --- | --- | --- |
| Hyperglycemia | 0 | 0.0% | --- | --- | --- | --- | --- |
| Arthralgia | 8 | 21.1% | 11.4 | 21.9 | 1 | 1 | 63 |
| Myalgia | 3 | 7.9% | 28.7 | 30.9 | 20 | 3 | 63 |
| Decreased appetite | 2 | 5.3% | 32.0 | 43.8 | 32 | 1 | 63 |
| Cardiomyopathy | 1 | 2.6% | 1.0 | --- | 1 | 1 | 1 |
| EKG abnormalities | 1 | 2.6% | 1.0 | --- | 1 | 1 | 1 |
| Hypertension | 0 | 0.0% | --- | --- | --- | --- | --- |
| Retinopathy | 0 | 0.0% | --- | --- | --- | --- | --- |
| Any AE listed above | 32 | 84.2% | 24.6 | 72.2 | 3 | 1 | 394 |
| None of the AEs listed above | 6 | 15.8% |  |  |  |  |  |
